# Supplementary material for: Novel adult cortical neuron processing and screening method illustrates sex- and age-dependent effects of pharmaceutical compounds
Source: Sci Rep. 2022 Jul 30;12:13125. doi: 10.1038/s41598-022-17389-4 (PMC9338961; doi:10.1038/s41598-022-17389-4)
Supplement: Supplementary file 1 — Supplementary Information. [file 41598_2022_17389_MOESM1_ESM.docx]

Supplementary data/info:

*Cell Culture Protocol*:

Final neuron isolation, processing, and plating procedure referred to as the ‘Modified Protocol’:

1. Fill each of the wells on the 384-well glass bottom plate (MGB101-1-2-LG-L Brooks Life Sciences or 781892 Greiner-Bio 384 Well SensoPlate™) being used with 18 µL of 50 µg/mL Poly-D-Lysine (PDL), then tap plate on the side 5 times to have the liquid settle on the glass surface
2. Incubate plate in 5% CO_2_ incubator set at 37 °C for 1-48 hours
3. After incubation, wash the wells with H_2_O 4× and set to dry at room temperature for 4-48 hours
4. After euthanization of mice, remove the brain from the head and place in cold Hank's balanced salt solution (HBSS) and microdissect region of interest
5. Fill each gentleMACS™ C Tube (Miltenyi Biotec, # 130-093-237) with 5 mL of 0.3 mg/mL papain (Worthington LS003126) and place up to 1.25 grams of brain tissue in each gentleMACS™ C Tube
6. Cut the brain tissue in the gentleMACS™ C Tube into small pieces using scissors and quickly invert, make sure all brain pieces are in solution while tube is upside down
7. Place tube on the gentleMACS™ Octo Dissociator with Heaters (Miltenyi Biotec, # 130-096-427) and attach the heating cuffs
8. Run the program “gentleMACS Program 37C_ABDK_01”
9. After program completion, strain the contents of the gentleMACS™ C Tube through a 70 µm cell strainer placed on top of a 50 mL conical centrifuge tube
10. Add 7 mL of cold Dulbecco's Phosphate-Buffered Saline with glucose and pyruvate (DPBS, Thermo Fisher Scientific, 14287072) into every gentleMACS™ C Tube used and add the contents into the respective 50 mL tube
11. Centrifuge the 50 mL tubes at 300×g for 7 minutes at 4 °C and aspirate supernatant completely afterwards
12. Make Debris Removal Solution: 1800 µL of Debris Removal Concentrate (Miltenyi Biotec, # 130-109-398) mixed in 6200 µL cold DPBS
13. Resuspend pellet in 8 mL Debris Removal Solution for every 1.25 grams of brain tissue
14. Mix contents of tube until all sample is resuspended and solution looks homogenous
15. Very slowly, add 4 mL of cold DPBS on top of the solution
16. Centrifuge at 4 °C and 3000×g for 5 minutes with slow acceleration and deceleration
17. Aspirate out all the top layer of clear liquid and any flat layers of myelin, leave the debris below the flat layer of myelin in the tube and minimize the amount of debris removal solution aspirated
18. Fill DPBS until the 50 mL mark
19. Centrifuge the 50 mL tubes at 300×g for 5 minutes at 4 °C and aspirate supernatant completely afterwards
20. Make Red Blood Cell Remover Solution: 125 µL Red Blood Cell Lysis Solution 10× (Miltenyi Biotec, # 130-094-183) mixed with 1125 µL of H_2_O
21. Resuspend each pellet in 1.25 mL of Red Blood Cell Remover Solution for every 1.25 grams of starting brain tissue
22. Incubate the tubes in the dark at 4 °C for 10 minutes
23. Make 0.5% bovine serum albumin (BSA) solution: 25 mL of MACS BSA Stock Solution (Miltenyi Biotec, # 130-091-376) mixed in 475 mL DPBS
24. Add 12.5 mL of 0.5% BSA for every 1.25 grams of starting brain tissue
25. Centrifuge at 300×g for 5 minutes at 4 °C and aspirate supernatant completely afterwards
26. Resuspend each pellet in 80 µL of 0.5% BSA and 20 µL of Non-Neuronal Cells Biotin-Antibody Cocktail (Miltenyi Biotec, # 130-115-389) for every 1.25 grams of starting brain tissue and transfer solution into 15 mL conical centrifuge tubes
27. Mix well, then incubate in the dark for 5 minutes at 4 °C
28. Add 1 mL of 0.5% BSA for every 1.25 grams of cortical tissue
29. Centrifuge at 300×g for 5 minutes at 4 °C and aspirate supernatant completely afterwards
30. Resuspend each pellet in 80 µL of 0.5% BSA and 20 µL of Anti-Biotin MicroBeads (Miltenyi Biotec, # 130-115-389)
31. Mix well, then incubate in the dark for 10 minutes at 4 °C
32. During this time, wash the LS column (Miltenyi Biotec, #130-042-401) by adding 5 mL of 0.5% BSA into it as it is attached to QuadroMACS™ Separator (Miltenyi Biotec, # 130-090-976)
33. Add 5 mL of 0.5% BSA into the 15 mL tubes after incubation time and mix gently
34. Transfer all liquid in 15 mL tube into the washed LS columns and collect liquid being expelled (using only the force of gravity) as the negative fraction (neurons)
35. Place the LS columns on new 15 mL tubes and add 5 mL of 0.5% BSA into LS column
36. Use the included plunger to quickly force the liquid inside the LS column into the 15 mL tube and collect as positive fraction (non-neuron neural cells)
37. Centrifuge the 15 mL tubes at 300×g for 5 minutes at 4 °C to pellet and resuspend neurons in 30 µL of neuron media

Unless noted, the neuron media consists of:

- 1. MACS Neuro Media (Miltenyi Biotec, # 130-093-570)
  2. 2 mM L-alanine-L-glutamine dipeptide (Sigma-Aldrich, G8541-100ML)
  3. 1× B-27™ Plus Supplement (ThermoFisher Scientific, A3582801)
  4. Optional: 50 units/mL of penicillin and 50 µg/mL of streptomycin (Corning, 30-002-CI)

1. Add neurons into each of the PDL coated wells
2. Place cells in 5% CO2 for the stated days *in vitro* (DIV)
